# Controlled Drug Release by the Pore Structure in Polydimethylsiloxane Transdermal Patches

**DOI:** 10.3390/polym12071520

**Published:** 2020-07-08

**Authors:** Barbara Mikolaszek, Jurgita Kazlauske, Anette Larsson, Malgorzata Sznitowska

**Affiliations:** 1 Department of Pharmaceutical Technology, Faculty of Pharmacy, Medical University of Gdansk, Hallera 107 Street, 80-210 Gdansk, Poland; bmiko@gumed.edu.pl; 2 Pharmaceutical Technology, Department of Chemistry and Chemical Engineering, Chalmers University of Technology, 412 96 Gothenburg, Sweden; jurgita.kazlauske@chalmers.se (J.K.); anette.larsson@chalmers.se (A.L.); 3 SuMo BIOMATERIALS, VINN Excellence Centre, 412 96 Gothenburg, Sweden; 4 AstraZeneca R&D Gothenburg, 431 83 Mölndal, Sweden

**Keywords:** silicone, transdermal patch, permeability, microstructure, drug release, indomethacin

## Abstract

The use of polydimethylsiloxanes (PDMS) as a drug carrier in transdermal adhesive patches is limited and there is insufficient data on the polymer structure and diffusivity, especially when additives modify the matrix. PDMS films with liquid additives (10% *w*/*w*): silicone oil (SO), polyoxyethylene glycol (PEG) or propylene glycol (PG) were prepared and indomethacin (IND; 5% *w*/*w*) was incorporated as a model active substance. The microstructure of the PDMS matrix and its permeability to water was investigated and correlated to the kinetics of the in-vitro IND release from the film. Three microscopic techniques were used to characterize in detail the microstructure of PDMS films: scanning electron microscopy, fluorescent microscopy and atomic force microscopy. PDMS films with hydrophilic PEG or PG showed different two-phase structures. A two-fold increase in steady-state flux of IND and increased water transport in the presence of PEG was attributed to the pore-like channels created by this polar solvent in the PDMS matrix. This effect was not observed in the films with PG, where only discontinuous droplet-like structures were visible. All additives significantly changed the tensile parameters of the films but the effects were not very pronounced.

## 1. Introduction

The transdermal drug administration route remains a constant focus of pharmaceutical research [1,2]. Transdermal patches are formulations applied to the skin for local or systemic drug delivery. In their development, selection of a matrix polymeric material determines further development stages and the dosage form final performance. Considering the demanding scientific and engineering challenges associated with the design of transdermal patches and the limited number of the active substances that permeate the skin barrier, the rather small contribution of these complex dosage forms to the market is understandable [3].

Polydimethylsiloxanes (PDMS) have been used in a number of medical applications, such as medical devices or wound care products, whereas their usage as a drug carrier in commercially available dosage forms is very limited. Being inert and biocompatible materials, PDMS are suitable for a matrix of formulations like transdermal patches, which provide a prolonged drug–skin contact, especially when these polymers are bioadhesive. Development of medical drug delivery patches require a number of technological considerations, many focused on the drug release kinetics from the dosage form to the skin surface [4,5]. Previously we explored the PDMS material as a film-forming excipient suitable for prolonged skin contact in scar treatment therapy [6,7]. In this work, we focused on better understanding the microstructure of the films and evaluation of its effect on permeability to water and the drug release process. This study provides a new insight on the process of drug diffusion through PDMS films, which could lead to potential applications in drug delivery technology.

Drug release from solid non-degradable polymeric matrices (cross-linked PDMS for instance) is controlled by the diffusion process, as stated by Higuchi (1961), and described by a simplified equation when the initial high excess of the drug (*c_ini_* >> *c_s_*) is present in the matrix [8,9]:

(1) $$Q_{t}=\sqrt{Dc_{s}t2c_{ini}}$$

where *Q_t_* is cumulative amount of the drug released at the time *t*, *D* is the diffusion coefficient of the drug in the matrix, *c_ini_*, is the initial drug concentration in the matrix and, *c_s_* is drug solubility in the matrix. The most common approach to enhance the release of the drug is to increase its solubility in the polymeric matrix, which can be achieved by addition of some excipients [10]. In this study liquid excipients, hydrophilic or lipophilic, were introduced to the PDMS matrix. The liquid components should modify the drug release kinetics by increasing drug solubility. Simultaneously, in the presence of additives, the matrix microstructure can be expected to change, also affecting its diffusivity.

We investigated whether the new inner structure, with increased porosity and hydrophilic domains, can be used as a tool for controlling the drug release, as it is expected that penetration of water into the modified polymer matrix is promoted. A similar approach was explored previously for PDMS scaffolds with a hollow pore architecture, where permeability–porosity correlation was found [11]. Relevance of the material porosity for water permeation and controlled drug release was often taken into consideration when different dosage forms were characterized. Maximizing the permeability for water with retained mechanical durability is usually the goal in development of fast disintegrating tablets or extended-release coated tablets [12,13,14]. More sophisticated control of the drug release is usually expected for microspheres, implants or hydrogels, where swelling additionally contributes to the diffusion mechanism [15,16,17]. Although the approach of matrix modification with solid or liquid hydrophilic excipients to increase water transport or drug release was reported by other authors [18,19], the pore formation in PDMS materials suitable for transdermal drug delivery has been an unexplored topic.

The relationship between the composition, structure and performance of the dosage form is often bypassed in favor of the simpler composition–performance correlation, which in turn does not provide the necessary data to understand the process and rationally optimize the drug delivery formulation. Even though in-vitro release tests remain a key tool in dosage form development, the dissolution profile can provide limited insights into the diffusion mechanism. The integration of imaging techniques with dissolution tests and water transport studies may be a viable approach to advanced formulation development [12].

Thus, in this work, structural changes in the PDMS matrix by liquid additives which also simultaneously increase drug solubility in the matrix has been explored, in order to search for methods to improve the drug diffusion and release from the transdermal patch. The lack of such knowledge makes development of these formulations time-consuming, with much experimental work required. This new insight should contribute to the rational design and optimization of PDMS-based matrices that can provide controlled release in transdermal therapy.

In the present study indomethacin (IND), an anti-inflammatory drug, was used as a model drug molecule. Not without significance is the fact that the administration of indomethacin in the form of a transdermal patch is an attractive alternative to analgesic ointments.

## 2. Materials and Methods 

### 2.1. Materials

A two-part kit of platinum-catalyzed silicone elastomer, Gumosil AD-1 (liquid, two-component A + B, cross-linked by platinum catalyst polydimethylsiloxane, PDMS) was purchased from Silikony Polskie (Nowa Sarzyna, Poland) and used as a solid matrix former. Silicone oil SO (Q7-9120, kinematic viscosity of 350 cSt; Dow Corning, Wiesbaden, Germany), or polyethylene glycol PEG (M_w_ 300; Sigma-Aldrich, Steinheim, Germany) and propylene glycol PG (Sigma–Aldrich, Steinheim, Germany) were used as hydrophilic additives. Indomethacin IND (Sigma–Aldrich, Steinheim, Germany) was a model drug. Tritium-labelled water ^3^H_2_O (37 MBq/g, 18 g/mol) and scintillation cocktail Opti-phase Hisafe 3 were purchased from Perkin Elmer (Walthman, MA, USA). All other reagents and solvents were of analytical grade and purchased from J.T. Baker (South Plainfield, NJ, USA). If needed, Milli-Q purified water was used (Millipore, Merck, Germany).

### 2.2. PMDS Films Preparation

Both drug-free films and drug-loaded films were made using a casting technique described previously [7]. In brief, the preparation procedure started with blending indomethacin (IND) (5% *w*/*w* of a final mass of the films) with a liquid additive: SO, PEG or PG (10% *w*/*w*, the mixing ratio 1:2). In order to achieve a homogenous particle distribution, a high torque mixer (RZR 2021, Heidolph Instruments, Schwabach, Germany) equipped with a PFTE stirring rod was used (Carl Roth, Karlsruhe, Germany). The blend was added to part A of Gumosil AD-1 and mixed at 240 rpm for 5 min. Then, the component B was added and mixed at 120 rpm for 2 min. For Gumosil AD-1 parts A and B the mixing ratio was 9:1, by weight. For the drug-free films the liquid additive was mixed directly with component A of PDMS, then the procedure was continued as described above. Finally, the mixture was de-aerated in a high vacuum and cast onto a glass plate to form a film of 500 μm ± 15 μm in thickness. For the water permeation experiment, thinner films were obtained (approx. 100 μm thick). The curing process (cross-linking of PDMS) was conducted at 23 ± 1 °C for 24 h in a high vacuum. The prepared films were packed into polyethylene bags with a tight zipper closure and stored in a controlled environment (25 ± 1 °C, RH 60%) for 7 days before analysis.

The thickness of the obtained films was accurately measured with an infrared gauge MiniTest 730 (Electro Physik, Koln, Germany) and expressed as an average (*n* = 100), further confirmed with the cross-section observation with an optical microscope. Density of the films (ρ) was calculated considering the weight and thickness, for at least three samples (expressed in mg/cm^3^).

### 2.3. Fluorescent Microscopy

To assess the uniformity of particle distribution and to quantify the volume fraction of the particles in the films, microscopic images of the films’ cross-sections were obtained using a fluorescent microscope (Nikon Eclipse i50, Nikon Instruments, Tokyo, Japan) with an excitation filter: 330–380 nm and emission filter: 400 nm. The imaging study was conducted with Zeiss objectives: 10× for a general overview of the sample and Ph2 DLL 40× magnification lens to obtain the images within in-depth range of 15 μm. The images that have been captured in a different axial (z) dimension were processed with NIS Elements Advanced Research 3.20 software (Nikon Instruments, Tokyo, Japan). First, a deconvolution algorithm was used to preliminary eliminate blurs on the single picture and to create an all-in-focus image with the Extended Depth of Focus (EDF) module, which resulted in virtual 3D images. Before further image analysis, the thresholds were defined for enabling separation of the particles intensity from the background. IND particle size and volume of the solid IND fraction in the analyzed films (volume fraction of the particles in PDMS matrix) were measured and calculated with NIS software.

### 2.4. Scanning Electron Microscopy

Different structures of PDMS films were studied by a field emission scanning electron microscope (SEM) (Leo Ultra 55 FEG SEM, LeoElectron Microscopy, Cambridge, UK), by in-line detection mode at 3 kV. Examination of cross-sections of the films and their surfaces, was performed. For cross-section observations, the films were cut with a microsurgical knife, in a way that ensured that the pore structure was retained. Prior to the microscopic observations, thin slices of approx. 0.5 mm were mounted on conductive carbon adhesive tabs and coated with a thin layer of gold in an ion sputtering device.

### 2.5. Atomic Force Microscopy

AFM (atomic force microscopy) experiments were performed using a Flex-Axiom AFM equipped with an ATS 204 translation isostage (Nanosurf AG, Liestal, Switzerland) and C3000 controller in tapping mode under ambient conditions. The AFM probe, a PtIr5 coated cantilever (PPP-EFM, Nanosensors, Neuchatel, Switzerland) was used for imaging with nominal force constants between 0.5 and 9.5 N/m and resonance frequencies between 45 and 115 kHz. Topography, amplitude and phase data were collected. A large scan of 60 μm × 60 μm area of each formulation was made, then at least 3 images of 15 μm × 15 μm dimensions were further obtained for roughness analysis. Scans in Z-axis geometry were analyzed using Nanosurf (version 3.8.0.8, Nanosurf AG, Liestal, Switzerland) and Gwyddion (version 2.49, Brno, Czech Republic) software for image processing.

### 2.6. Mechanical Testing

The tensile properties of the films were determined by a TA.XT plus texture analyzer (Stable Micro Systems, Godalming, United Kingdom). Tensile grips in a tension mode were used to obtain stress–strain curves. The initial distance of grips was 30 mm, whereas other test parameters were set as follows: tensile extension 1 mm/s, loading cell 5 kg, detection limit 0.049 N. Measurements were performed after equilibration of the samples for 24 h at 24 ± 1 °C and relative humidity of 35 ± 5%.

In order to describe elasticity and durability of the formulated patches, tensile strength (TS, MPa), elongation at break (% EB) and Young modulus (Modulus E, MPa) parameters were determined from the obtained stress–strain curves with Exponent software (version 6.1.12.0, Stable Micro Systems, Godalming, United Kingdom), described previously in details [7].

### 2.7. Estimation of Drug Dissolved Fraction

To estimate the fraction of IND dissolved in the silicone-based films (DF), its solubility in all liquid additives was examined and compared with the published data. Reported solubility for IND (at 25 °C) are as follows: 0.01 ± 0.004 mg/mL in SO, 95.3 ± 0.05 mg/mL in PEG and 116.3 ± 0.07 mg/mL in PG [20,21]. The solubility of IND in silicone oil was used as a surrogate for its solubility in PDMS film, which is an approach well established in practice [22]. Therefore, the dissolved fraction (DF %) of the total amount of IND incorporated in the film was estimated by equation:

(2) $$DF=\frac{C_{PDMS}\times V_{PDMS}+C_{LA}\times V_{LA}}{m_{ind}}\times 100$$

where *C_PDMS_* and *C_LA_* are the solubility of IND in PDMS and in a liquid additive—LA (SO, PEG or PG) at 25 °C [mg/mL], respectively, *V_PDMS_* and *V_LA_* are the volumes of liquid additives [ml], and *m_ind_* is a total amount of IND in the formulation [g]. The assumption that saturation of the solute was achieved in all phases was made.

### 2.8. Swelling and Erosion

PDMS film samples (0.65 cm^2^) were weighed (initial mass), placed in a wire sinker (see Section 2.10) and transferred to a beaker with 100 mL water at 37 °C ± 0.5 °C (water bath with a shaker at 135 rpm). At the predetermined time points the films were withdrawn from the fluid and any residual water from the surface was dried with a dust free paper cloth. The film sample was weighed accurately. When no mass gain over 3 time points was noted (maximal mass), the sample was dried in an oven at 60 °C until loss of the film mass achieved the plateau. The minimal film mass value was marked as the final mass. Degrees of swelling and erosion of each film at the given time points were calculated according to the equations:

(3) $$Swelling\;degree\;\left[\%\right]=\frac{maximal\;mass\;\left[g\right]-initial\;mass\;\left[g\right]}{initial\;mass\;\left[g\right]}\times 100$$

(4) $$Erosion\;\left[\%\right]=\frac{initial\;mass\;\left[g\right]-final\;mass\;\left[g\right]}{initial\;mass\;\left[g\right]}\times 100$$

After the test, the samples were freeze-dried and their cross-sections were examined by SEM. Since IND solubility in water is very low (0.0025 mg/mL), the weight loss due to its dissolution during the experiment was ignored.

### 2.9. Water Permeation

Permeability measurements for the drug-free and drug-loaded films were performed according to the experimental setup described in details by Andersson et al. [13]. The experiment was conducted in ambient conditions. The square film samples (2.25 cm^2^) were examined for their integrity and the thickness was confirmed. The sample was placed in a horizontal cell, between two chambers (donor and acceptor compartments), 15 mL of deionized water was added to each compartment and then the cell was transferred to a thermostated shaker (100 rpm). After equilibration, a tritium-labelled water (10 μL, 370 kBq) was added to the donor compartment. At the specified time intervals 500 μL samples from the acceptor compartment were withdrawn and replaced with water, then mixed with the scintillation cocktail. The collected samples were analyzed in a Liquid Scintillation Analyser TriCarb 2810 TR (ElmerPerkin, Waltham, MA, USA). The experiment was conducted until a steady state flux was achieved (up to 7 days). The scintillation counts measured in the acceptor compartment was stated to be proportional to the diffused water mass, hence the mass transfer rate was calculated (*m*) from the steady-state slope. The permeability (*P*_LS_) value was determined from the linear regression and calculated from the equation:

(5) $$P_{LS}=\frac{m\times h}{A\times \left(C_{D}-C_{A}\right)}$$

where *h* is a film thickness, *A* is the area of a film available for diffusion and *C_A_* and *C_D_* are average concentrations of tritium-labelled water in the acceptor and donor chambers, respectively.

### 2.10. In Vitro Drug Release

An in-vitro dissolution test was performed with assurance of the sink conditions, employing the method described before [6]. The tests were conducted for IND-loaded silicone films (3.5 mg/cm^2^) cut in squares (surface area 9 cm^2^). A 100 mL volume of phosphate buffer (pH 7.4 at 37 °C ± 0.5 °C) was used as an acceptor fluid (water bath with a shaker at 135 rpm). To prevent film floating during the experiment, a wire sinker was used. The experiment was conducted for 48 h. In order to achieve the sink conditions, every 12 h the entire volume of acceptor medium was replaced with an equal volume of the fresh medium. The samples of the acceptor medium were assayed for IND concentration at 320 nm by UV/Vis spectroscopy (Jasco V-530, Jasco, OK, USA) method within the calibration range of 5–50 μg/mL.

### 2.11. Statistical Data Analysis

The results were expressed as a mean ± standard deviation. When appropriate, the obtained data were compared and analyzed by parametric multi-way ANOVA with post-hoc comparisons with Statistica (version 12.0, StatSoft, Tulsa, OK, USA). For Parson’s correlation analysis R (version 3.2.5., R Foundation for Statistical Computing, Vienna, Austria) was used. In all cases, *p* < 0.05 denoted significance, unless otherwise indicated.

## 3. Results and Discussion

In our previous work, it was shown that PDMS patches have unique features that can be useful for the design of dermal drug delivery carriers [6,7]. The present study explored the possibility of modification of PDMS films to enhance the release of a model drug—IND. Since many physicochemical features can affect the drug release mechanism, a multiple approach was considered essential. Firstly, the study focused on increasing the release rate by drug solubility enhancement in the matrix. PEG, PG and low-viscosity SO were chosen as solvents, hence dissolved fraction of IND in both liquid and solid PDMS component was assessed. Additionally, the liquid additives were supposed to alter the inner structure of a solid PDMS matrix. Estimation of the relevance of each of these factors for transdermal patch formulation performance was conducted.

The combination of three microscopic techniques was used to characterize in details the microstructure of PDMS films. SEM was used to provide a general overview of the film morphology, whereas fluorescent microscopy was employed to determine the distribution of IND within the polymeric film and assess the size of IND particles. AFM was used to more closely investigate the two-phase microstructure created by the liquid additives. Apart from the drug release experiments, water transport in the PMDS films modified with the additives was evaluated in order to characterize diffusion dynamics.

### 3.1. Fluorescent Microscope Imaging

Fluorescence microscopy with digital image analysis allowed a clear overview of the suspended IND particles without interference from the PDMS background, since the liquid additives caused no visible changes in PDMS fluorescence microscopic image.

**Figure 1:**
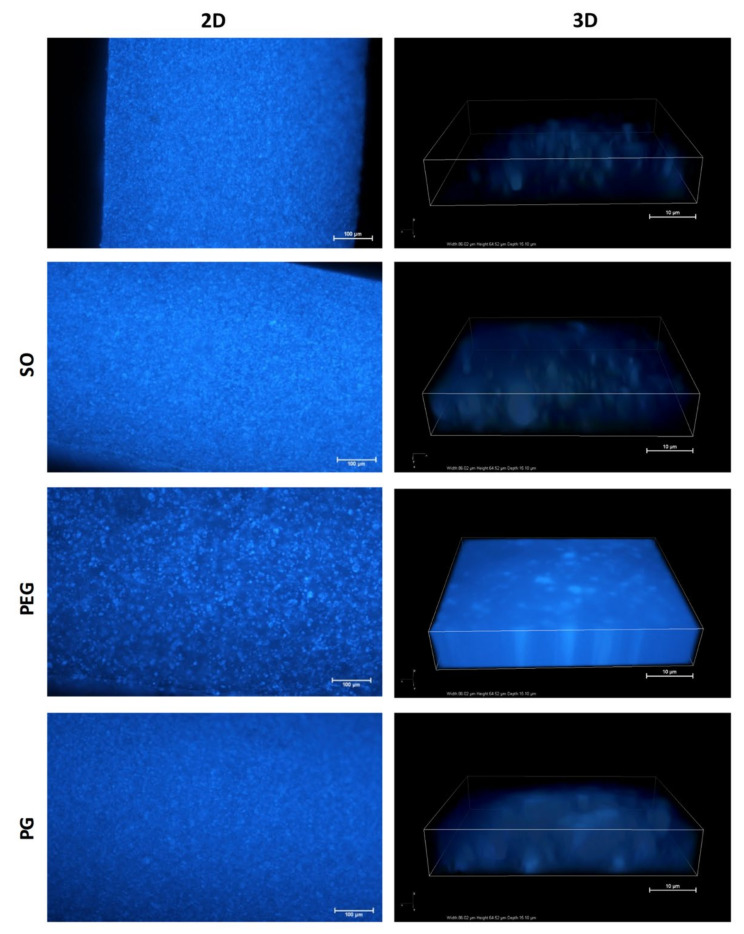
Fluorescence microscopy images of cross-section of polydimethylsiloxane (PDMS) films with indomethacin (IND): X—additive-free film, SO—with silicone oil, PEG—polyethylene glycol or PG—propylene glycol. Left column—2D sample overview (scale 100 μm), right column—3D in-depth images (scale 10 μm).

Figure 1, in the left column, shows microscopic images of the cross-section of the additive-free films and the films with one of the liquid additives, all loaded with IND (5%).

Preliminary examination at 10× magnitude confirmed the uniform distribution of IND particles across the full-thickness films in all PDMS blends. SO, PEG and PG added at the first stage of film preparation allowed elimination of particle aggregates visible in the images of the additive-free formulation (up to maximum size of 10 μm). Therefore, the liquid additives allow a homogenous distribution of the suspended particles within the matrix if the proposed compounding protocol is applied. Table 1 presents a comparison of IND particle size in the powder used for film preparation and in PDMS films. No significant differences in the mean particle size was found for the examined blends.

In case of PEG and PG films an increased light intensity from a matrix was considered to be the effect of IND, which dissolved in the matrix (Table 1). Further analysis of the acquired 3D images (Figure 1, right column) allowed assessment of the particle volume fraction within the film and estimation of whether the liquid additives affect the size and number of IND particles in the PDMS films. A clear difference, depending on the additive type, was observed (Table 1). Approximately 30% of the analyzed volume of PDMS and PDMS–SO films was identified as IND particles. Significant decrease of the solid particles volume (down to 7%) was noted for both hydrophilic excipients (PEG and PG), which explained the previous observation of the amplified matrix luminescence. To further investigate the observed phenomena, experimental data were correlated with the theoretically estimated fraction of IND dissolved in the films (DF), where calculation was based on IND solubility in the additives and in PDMS. DF value for the SO containing film was found comparable to the additive-free film, which agrees with the particle volume fraction and confirms similarity of those formulations in terms of IND distribution. Theoretical estimation of IND fraction dissolved in the additive (DF) suggested, that a significant amount of the active substance can be present in the molecular state as dissolved in a hydrophilic phase of the films. This assumption is consistent with much lower fraction of the solid particles observed in the films with PG and PEG.

### 3.2. SEM Imaging

Scanning electron microscopy was used to study the films morphology. Generally, it allowed observation of the spatial distribution of the separated components and changes caused by the additives in the inner structure of the films.

**Figure 2:**
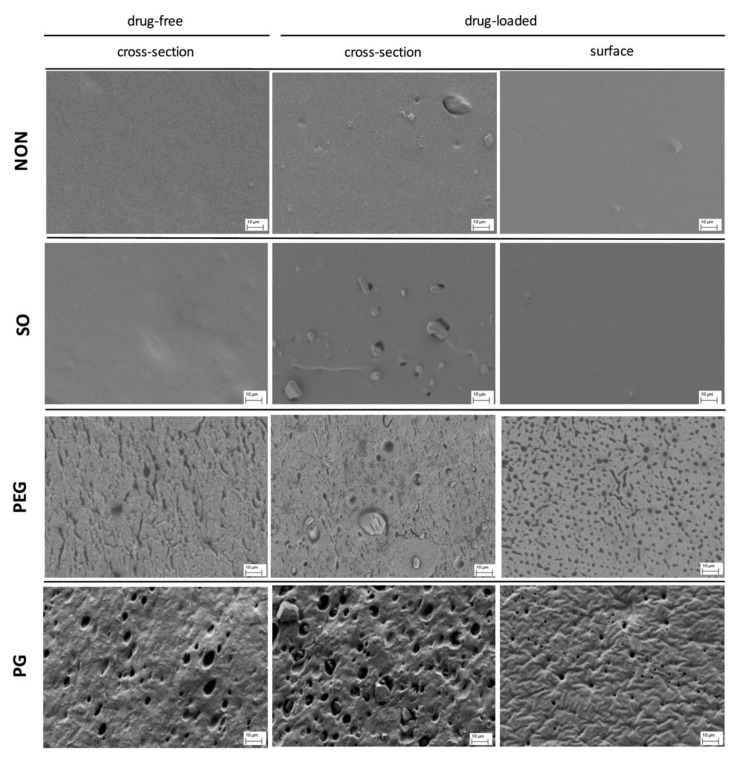
SEM images of the cross-section and the surface of PDMS films modified with liquid additives (scale 10 μm).

The most pronounced differences in the film structure were observed for the samples containing PEG or PG, where the pore-resembling structures were observed, whilst SO had no visible effect on the basic PDMS structure (Figure 2). IND particles were visible in drug-loaded films, with size resembling the mean size of the particles given in Table 1.

In the case of both hydrophilic liquid additives, the excipient-rich domains can be clearly distinguished. For PG film, a pin-hole-like (max. 2 μm in diameter) topography of the surface can be noticed, whereas the cross-section revealed a homogenous distribution of droplet-like structures of 5–10 μm in diameter. The microstructure formed within the network can be described as highly uniform and regular in the pore arrangement. In contrast, PEG-loaded film differed in both shape and size of the observed structures: more irregular, channel-shaped formations can be seen with only single droplets present (up to 5 μm). Further examination of the film surface has shown multiple droplets with a considerable number of particles with elongated shapes.

As expected, for the two examined hydrophilic excipients a phase separation in the patch was clearly visible. However, PEG was found to be the most pronounced pore-former regarding the pore shape and distribution. Since the additives generated diverse networks in PDMS, different properties of these films may be expected.

### 3.3. AFM Analysis

**Figure 3:**
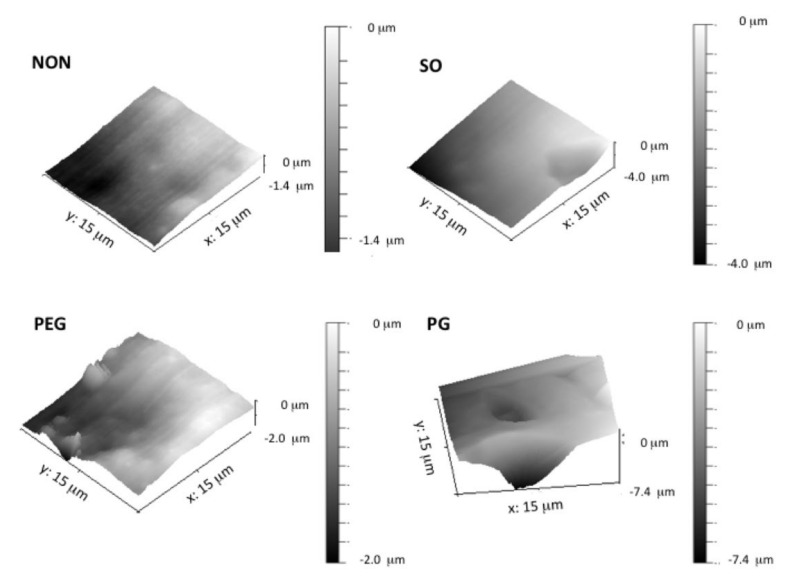
Atomic force microscopy (AFM) three-dimensional images of PDMS films with liquid additives and IND (different scaling of the z-axis).

To further investigate the two-phase structures created by the addition of hydrophilic liquid additives, atomic force microscopy was employed. Figure 3 presents an example of 3D images of cross-sections of the IND-loaded films. In each of the examined films, single IND particles were observed with sizes consistent with previous findings.

Roughness of the surface for the additive-free film was noted, which was contributed by the amorphous silica particles that are commonly added to silicone biomaterials as a reinforcing filler [4]. It can be seen that a slightly smoother plane was achieved with the addition of SO to the film. The texture of PG-loaded films was regular, and the presence of spherical droplets, already noticed in the corresponding SEM images, was confirmed. Since this dot-like structure was surrounded by smooth PDMS regions, this leads to the conclusion that PG droplets do not form a network but are homogenously distributed within the film. In contrast, within examined cross-sections PEG created rough and elongated irregular structures. Moreover, due to the presence of PEG, complex channel-like domains make a larger contribution to the highly heterogeneous structure, that can be seen in AFM (Figure 3), which might have not been concluded from SEM images alone.

### 3.4. Mechanical Characteristics of PDMS Films

Mechanical properties of the films were characterized by tensile strength (TS), Young modulus (Module E) and elongation at break (% EB) values presented in Table 2, together with the calculated density of the films. As suggested before, it was hypothesized that the matrix inner structure may influence the mechanical properties of the films profoundly. When the pure PDMS film was used as a reference, multiple effects were observed for composed films, with statistical significance of the differences confirmed, which varied depending on the additive type and the presence of active substance.

In the IND-free films, all of the examined liquid additives significantly lowered the tensile strength of the PDMS films. The most pronounced decrease was observed in the case of SO, in spite of the fact that no additional structural contribution of the additive was noted, as was observed for PG and PEG. A similar mechanism of interference in polymeric films that could confirm this assumption was found in literature [23,24]. The Young modulus parameter of PDMS film significantly increased in the presence of PEG (i.e., the film was stiffer). GP caused an opposite effect and more flexible material was obtained, comparable to SO films. Moreover, considering the opposite effects in the presence of PEG or GP and higher density of the PEG films, it was concluded that the unique shape of the microstructures within the PEG film is the main cause of the observed changes.

Further changes in the film performance were noticed when the active substance was present. In general, simultaneous addition of IND and one of the excipients enhanced the strength of the films, but only in case of SO was the increase statistically significant, yet still not as pronounced as in the additive-free film. It should be noted that a simultaneous decrease of % EB value in the presence of GP indicates that the material is more fragile, due to liquid droplets present in the matrix. This might be due to IND occurring in a dissolved form in the excipients phase, where fewer particles can strengthen the material and alter the shear transfer, hence acting as a filler reinforcing the material (DF approx. 20%, Table 1). Thus, the basic mechanical analysis confirmed the pronounced structure differences within the films observed by imaging analysis.

### 3.5. Water Transport—Permeability, Swelling and Erosion of PDMS Films

A detailed characterization of the structures in PDMS films was made with SEM and AFM, while the contribution of the pore microstructure to the permeability of the films was assessed based on the results of the tritiated water permeation through the films and their ability to swell and erode.

**Figure 4:**
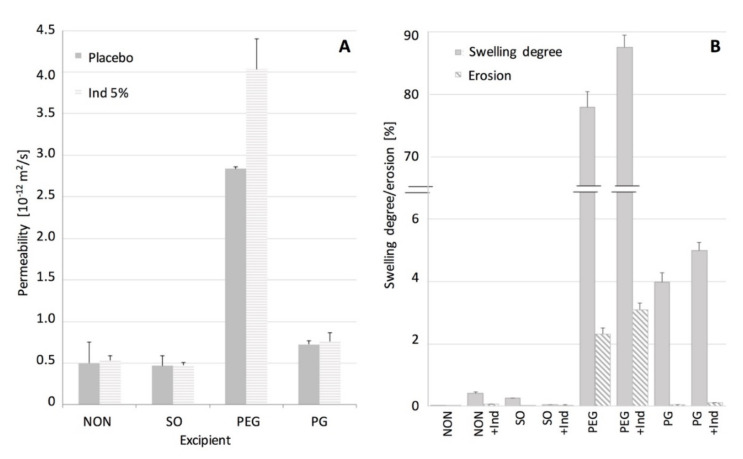
Water transport in PDMS films: (**A**) permeability of PDMS films with additives at steady state. (**B**) swelling and erosion degree (mean values ± standard deviation, *n* = 3).

In Figure 4. permeability of the examined films to water is shown, expressed as a steady state flux. The swelling and erosion data are also shown. When SO or IND were present in the films no changes in their permeability was noted, in contrast to PEG and GP films. It can be stated that in this case the water permeation will depend only on diffusivity of a hydrophobic PDMS material. Many authors stated that solid particles suspended in the matrix can increase the diffusivity of the polymeric materials [18,25], but in our case the phenomenon seemed unpronounced, probably due to relatively low IND particles load (5% *w*/*w*). This assumption was supported by similar effect described in the literature for a silicone elastomer [22]. Lack of swelling and erosion (below 0.5%) for films containing SO and IND indicated that the silicone polymer chains remained in the initial state, as noted for the pure PDMS. Thus it can be concluded that the water uptake and potential additive leakage do not interfere with the water flux.

The effect of the investigated hydrophilic excipients on the water transport through the films differed considerably (Figure 4). Although, in both cases, a statistically significant increase in the permeability to water was noted, PEG-containing films showed the more-pronounced improvement in water transport, with simultaneous substantial ability to swell (up to 90%). It was observed by Choi et al. [26] that water penetration into a hydrophobic material can be controlled by material porosity design. Regarding the microstructure of PEG films, it can be assumed that not only the size, but more the channel-like shape of the pores contributes to the water transport as additional paths, easily accessible for water, are created in the silicone structure. Since even higher permeability and swelling degree was noted in the presence of both PEG and IND, the solid particles in this case are likely to create additional regions or branching points in the pore structure, easily accessible for larger quantities of the medium to penetrate. Moreover, approximately 2.5% erosion of the film, further increased with simultaneous presence of IND, suggests the PEG leakage from the films, and seems to support observations on the branching of the PEG domains, where water has an access to deeper layers of the film and to the IND particles. Besides, it can be presumed that PEG is partially trapped in the pores it created, therefore not all of the regions are equally accessible to water, which explains a relatively low erosion of these films (lower than initially expected). Presumably, due to high molecular weight of PEG and differences in chemical structure of the surrounding silicone, PEG chains can be partially immobilized by PDMS surface free energy, as suggested by other authors [19].

Even though PG-containing films showed a porous inner structure, its effect on the permeability and material ability to swell, was much less manifested than in the case of PEG. Considering the low erosion of PG films, even those with IND, one can assume that water access to the inner part of the film seems to be limited by more isolated PG regions in the inner structure of the PG–PDMS films.

**Figure 5:**
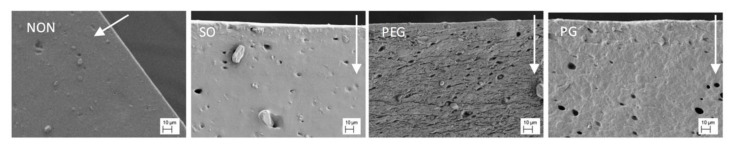
SEM images of cross-sections of PDMS films with IND and liquid excipients—freeze-dried films after exposure to water (scale 10 μm). Arrows indicate water flow direction.

To provide additional data, SEM study of the films exposed to water was conducted and the images are presented in Figure 5. The films soaked in water were freeze-dried before SEM examination of the cross-section. The pore structure in these films was compared with the corresponding untreated samples (Figure 2). For pure PDMS films and the films with SO no changes in the morphology were noted. After exposure to water the inner structure of PG-containing films revealed a lower concentration of PG droplets near the surface, undoubtedly due to being superficially washed out. The low water penetration into this film explains its minimal erosion and the swelling occurring only on the surface. Since the water uptake in PEG-loaded films was the highest one noted, in consequence the most pronounced changes in the microstructure were observed. In this case, the matrix seemed to shrink and vertical layers can be distinguished across the film, indicating where the film swelled and then eroded most significantly, with a clearly noticeable decrease of the characteristic channel structure.

**Figure 6:**
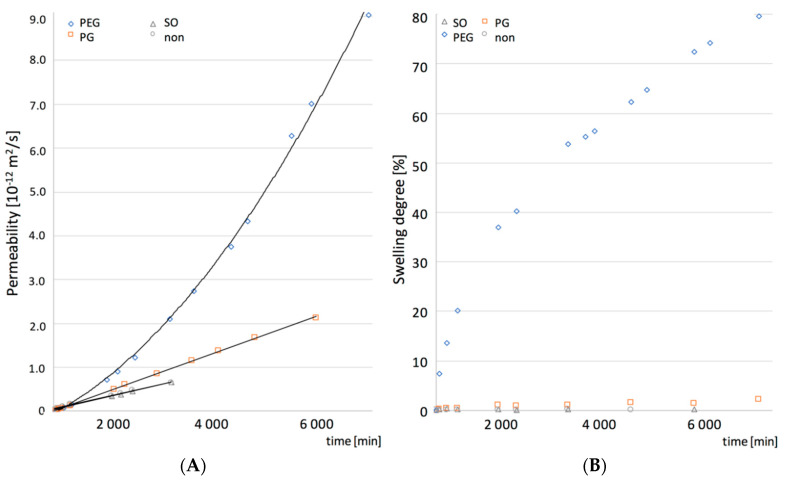
Permeability (**A**) with the corresponding swelling (**B**) curves as a function of time for PDMS films (non) and films with the additives SO, PEG or PG (mean values ± standard deviation, *n* = 3).

To further explain the observed structural phenomena and assess whether the water transport kinetics correlate with the swelling, more detailed analysis of the water permeability and the swelling kinetics was conducted and the results are presented in Figure 6. The additive-free PDMS, SO and PG films showed linearity in both the water permeation and the swelling slopes, resulting in a steady state flux observed during the course of experiment. In the case of water permeation through PEG-containing PDMS, two regions of the curve with the steady-state flux could be distinguished, which suggested a more complex mechanism of the water uptake. Moreover, a dual stage swelling rate was obtained, which seemed consistent with the film permeability.

The observed correlation between the water permeability and the swelling kinetics of the PDMS films with additives was further analyzed for its significance with the Pearson’s correlation coefficients factor (r_p_, Table 3). In the presence of hydrophilic excipients (PEG and PG) a strong positive correlation was found (r_p_ > 0.9, *p* < 0.05). However, for PDMS and SO-PDMS films, a poor correlation was found (r_p_ < 0.21, *p* < 0.05). These observations are considered as a support of the hypothesis that the increased permeability of PDMS films is attributed to the unique pore shape created by PEG or PG within the PDMS film.

### 3.6. IND Release Rate

**Figure 7:**
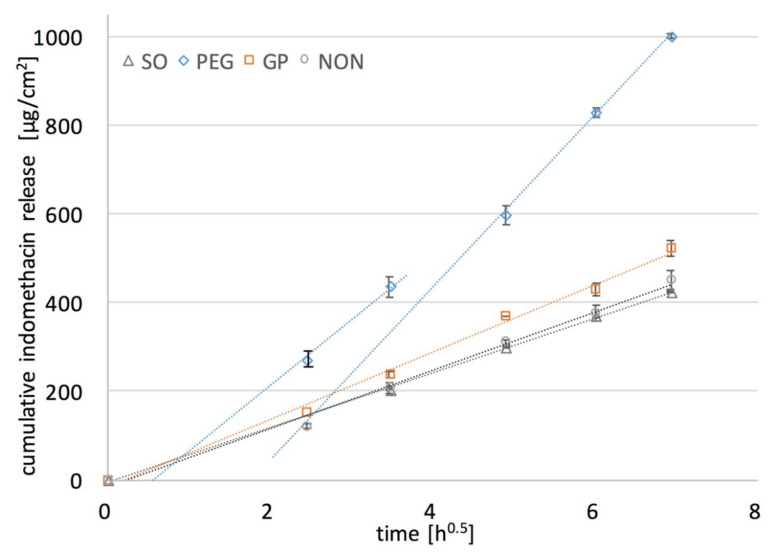
Effect of liquid additives (10% *w*/*w*) on the in-vitro release of IND (5% *w*/*w*) from PDMS films (mean ± standard deviation, *n* = 4) (symbols: experimental results for PDMS without and with additives: SO, PEG, PG).

The rate at which the active substance is released from a drug delivery matrix system can be controlled by a number of phenomena, among which most commonly mentioned in the literature are: water penetration into the system, polymer swelling and dissolution, drug diffusion through polymeric networks and the process of drug dissolution [27,28], all considered in this research so far. As proposed by Gehrke et al. [19], the importance of drug solubility in the matrix for reaching the optimal release kinetics should be taken into account. Thus, the most reasonable approach to optimize the release rate of an active substance in a transdermal patch is to increase the amount of the drug dissolved in the dosage form, which was achieved in this study by the addition of the liquid additives, PG and PEG. To evaluate this approach, the release of a model drug from PDMS films was examined. In vitro dissolution testing of the films containing 5% *w*/*w* of IND was performed in sink conditions, with an excess of the substance suspended in the formulation to sustain an infinite dose, both essential to obtain the insight into the mechanism of the release process [9]. The results expressed as a cumulative amount of IND released (µg/cm^2^) in a function of squared root of time are given in Figure 7.

In all of the examined formulations within the time of experiment (48 h) only a small fraction of the initial drug loading was released (up to 12% of IND). Comparable release rates were observed for PDMS films with SO and with no additives. The increase in the release rate of IND was noted only if PEG or PG was added to the film, but much higher values for PEG than for PG were found. This might be attributed to the more pronounced erosion and PEG leakage from the films upon exposure to the medium. It was very important that a considerable amount of IND was dissolved in PEG. Even though DF factor is much higher for PG than for PEG films (Table 3), the dissolved IND is located in the PG droplets distributed in the inner part of the film, and inaccessible to the dissolution medium. Therefore, the relatively low increase in the dissolution rate is caused only by the fraction of IND located near the surface of the film, since water penetration into the film deeper layer is rather limited. This presumption is supported by the observations with SEM (Figure 5).

Since the analyzed films were considered as a monolithic solid material with slab geometry (planar system), and IND solid particles uniformly dispersed in the PDMS, Higuchi kinetics behavior was expected [29]. In the first step of the analysis, the experimental data were fitted to the Higuchi model. Values of the dissolution constant at steady state flux (*k_s_*) and the determination coefficient (*R*^2^) indicating the agreement with the model are presented in Table 3, additionally shown graphically as dotted curves in Figure 7.

Based on the calculation, the release curve was linear for the reference PDMS film and the films containing PG or SO (*R*^2^ > 0.99) and for these formulations the drug release as a diffusion process based on the Fick’s first law was suggested. Considering the previous findings on water penetration kinetics in the films it can be concluded that the basic drug transport is controlled by the diffusivity of the PDMS polymeric structure. Nonetheless, it should be pointed out, that the presence of PG slightly improved the film performance (higher *k_s_* value), which is probably due to the superficially located drug dissolved in PG domains where additional dose of IND is present, and causes the increase of drug release at the beginning of the experiment. Regarding the observed rather minor increase in the in-vitro release rate from PG-containing films, together with the low erosion of the films occurring only on the surface (SEM), and small penetration of water, it can be stated that in the case of PG–PDMS films the drug diffusion is dominated mostly by diffusivity of the PDMS matrix.

In contrast, PEG–PDMS films substantially deviated from the Higuchi model. Interestingly, further calculations revealed that only the second part of the steady state slope (after 24 h of the release) was in good agreement with the Higuchi model (*R*^2^ > 0.98), which was also found to be consistent with the steady state water flux through the films observed in the permeation and swelling experiments. Thus, the assumption on the dual mechanism of drug release was stated, where not only solubility of the drug in the films but also the inner microstructure may affect the diffusion substantially. The correlation observed between the swelling and permeability kinetics and the release rate suggests that in the first stage a unique porosity of the matrix caused the increased water penetration into the film. As a result, drug-rich PEG was leached through the channels upon exposure to the dissolution medium. This can further be supported by the significant erosion of the films. The steady state of the release process was reached when the channels were fully filled and the contact surface between the medium and PDMS swollen pore network was constant. On this stage, when the drug flux fitted the Higuchi equation, the release was determined mostly by PDMS diffusivity.

## 4. Conclusions

The liquid additives, PEG and PG, were chosen in order to enhance drug release rate from the PDMS films and a multiple approach analysis was conducted to elucidate the structure of the film, which is important for the mechanism of the drug release process.

Silicone matrix was compatible with both hydrophilic additives and only minor changes in the tensile properties of the films were observed in comparison to the films containing a lipophilic SO liquid. The enhancement of IND release from the films containing PG or PEG was demonstrated, while the water permeation and swelling experiments have provided complimentary information on the mechanism of the drug release. A significant fraction of IND (up to 22%) was dissolved in the liquid phase, which contributed to the faster in-vitro release. The microscopic structure of PDMS films characterized in detail by SEM and AFM allowed explanation of the different effects of the liquid additives on the drug release rate and permeability to water. The enhanced drug release from the PDMS films containing PEG (two-fold increased steady-state flux) was attributed to the presence of hydrophilic pore-like channels. In contrast, PG created in the silicone matrix discontinuous droplet-like structures, thus its effect on the diffusivity of the silicone film was only moderate. Therefore, one can conclude that incorporation of a carefully selected hydrophilic excipient, capable of creating specific microstructure in the PDMS-based formulation, is a valuable approach, resulting in an enhanced drug release, and might be considered as a useful tool for controlling the dosage form performance. The conclusion is especially valuable in terms of the future development potential of PDMS-based transdermal patches.

## Figures and Tables

**Table 1:** The effect of liquid additives on IND particles characteristics in PDMS films (mean ± standard deviation, *n* > 500).

Formulation	Particle Size (μm)	Particles Volume Fraction (%)	DF (%) Calculated
IND powder	2.9 ± 4.3	-	-
PDMS_IND	2.2 ± 4.5	29.8 ± 1.5	0.24
PDMS_SO_IND	1.4 ± 6.1	30.9 ± 2.3	0.17
PDMS_PEG_IND	4.0 ± 5.5	6.8 ± 0.5	17.09
PDMS_GP_IND	0.6 ± 3.0	6.7 ± 0.3	22.63

**Table 2:** Effect of liquid additives and IND on PDMS films density (ρ, g/cm^3^) and tensile properties: tensile strength (TS), elongation at break (%EB), elastic modulus (Module E) (mean ± standard deviation, *n* > 10). Statistical significance: increase (+) or decrease (−) in comparison to the corresponding drug free/additive free formulation (significant differences are marked with bold fonts).

	Tensile Properties			Density ρ
Formulation	TS (MPa)	% EB	Module E (MPa)	(g/cm^3^)
PDMS	**2.79** **±** **0.53**	**310** **±** **43**	**0.55** **±** **0.03**	**1.706** **± 0.03**
PDMS_IND	3.60 ± 0.27 (+)	349 ± 36	0.59 ± 0.06 (+)	1.725 ±0.06
PDMS_SO	2.12 ± 0.38 (−)	302 ± 23	0.35 ± 0.03 (−)	1.719 ±0.10
PDMS_SO_IND	2.87 ± 0.28 (+)	314 ± 23	0.49 ± 0.05 (+)	1.730 ±0.07
PDMS_PEG	2.38 ± 0.31 (+)	232 ± 29 (−)	**0.77** **±** **0.07 (+)**	**2.093** **± 0.60**
PDMS_PEG_IND	2.35 ± 0.29 (−)	243 ± 26	**0.69** **±** **0.05 (−)**	**2.100** **± 0.08**
PDMS_GP	2.20 ± 0.21 (−)	240 ± 10 (−)	0.40 ± 0.01 (−)	1.804 ± 0.06
PDMS_GP_IND	2.22 ± 0.18 (−)	260 ± 20	0.38 ± 0.05 (−)	1.794 ± 0.04

Numbers were bolded for better data comparison.

**Table 3:** The effect of the additives on the swelling and permeability correlation coefficients and the release kinetics of IND (5%) from PDMS films.

PDMS Additive	Swelling and Permeability Coefficient ^1^		IND Release Kinetics Parameters ^2,3^		
	*r_p_*	*p*	*k_s_*	t*_s_*(h)	R^2^
NON	0.145	0.023	64.9	0–12	0.9974
SO	0.206	0.041	61.1	0–12	0.9991
PEG	**0.941**	0.032	**144.3**	**24–36**	**0.9817**
GP	0.911	0.016	75.4	0–12	0.9901

^1^ Pearson’s correlation coefficients factor (*r_p_*), *p* < 0.05 denotes significance, ^2^ Higuchi dissolution constant at steady state flux (*k_s_*), estimated time (t*_s_*) of steady state and determination coefficient (R^2^), ^3^ PEG values of the calculated fit to the Equation (1), when steady state flux was achieved. Numbers were bolded for better data comparison.
